# Potential of herbal formulas and bioactive metabolites in treating atherosclerosis: targeted modulation of macrophage polarization

**DOI:** 10.3389/fphar.2025.1631274

**Published:** 2025-11-06

**Authors:** Shixin Liu, Jinpeng Jing, Yunsha Zhang, Junchao Sun, Chaojun Zhu

**Affiliations:** 1 Graduate School of Tianjin University of Traditional Chinese Medicine, Tianjin, China; 2 Tianjin University of Traditional Chinese Medicine, Tianjin, China; 3 TCM Institute of Sore and Ulcer, Tianjin University of Traditional Chinese Medicine, Tianjin, China; 4 Tianjin Institute of Traditional Chinese Medicine Surgery, Tianjin, China; 5 The Second Affiliated Hospital of Tianjin University of Traditional Chinese Medicine, Tianjin, China

**Keywords:** atherosclerosis, macrophage polarization, signaling pathways, bioactive metabolites, herbal formulas, research progress

## Abstract

Macrophage polarization plays a pivotal role in the pathogenesis and plaque stability of atherosclerosis (AS). In response to microenvironmental cues, macrophages differentiate into pro-inflammatory M1 or anti-inflammatory M2 phenotypes, which respectively exacerbate or mitigate inflammatory responses and influence plaque progression. Emerging evidence highlights the therapeutic potential of targeting macrophage polarization through signaling pathways such as Toll-like receptor 4 (TLR4)/nuclear factor-kappa B (NF-κB), peroxisome proliferator-activated receptor γ (PPAR-γ), Janus kinase (JAK)-signal transducer and activator of transcription (STAT), phosphoinositide 3-kinase (PI3K)/protein kinase B (Akt) pathway, and mitogen-activated protein kinase (MAPK) pathway. Bioactive metabolites derived from traditional Chinese medicine (TCM)—including ginsenosides (e.g., Rb1, Rg3), berberine (BBR), curcumin (CUR), and tanshinone IIA (Tan IIA)—as well as herbal formulas like Bu Yang Huan Wu Decoction (BYHW) and Zhuyu Pill (ZYP), have demonstrated efficacy in promoting M2 polarization and suppressing M1 phenotypes, thereby attenuating AS. This review critically synthesizes the current body of evidence, with a primary focus on preclinical studies (*in vitro* and *in vivo*), which comprehensively synthesizes evidence on the targeted modulation of AS-associated macrophage polarization by bioactive metabolites and herbal formulas, with a unique emphasis on the role of TCM as a multi-target regulator of macrophage plasticity. This approach provides novel perspectives for the prevention and treatment of AS.

## 1 Introduction

Atherosclerosis (AS) is a chronic, progressive inflammatory disease of the arterial wall and is the pathological basis of cardiovascular, cerebrovascular, and peripheral vascular diseases, posing a major global health burden (He et al., 1999; Nedkoff et al., 2023). Its progression is driven by dysregulated lipid and glucose metabolism, hypertension, and lifestyle-related factors such as poor diet, smoking, and physical inactivity (Poznyak et al., 2020; Lechner et al., 2022). These risk factors collectively promote vascular calcification, loss of elasticity, and luminal narrowing. Central to disease progression are endothelial dysfunction, lipid retention, and chronic inflammation, which form a self-amplifying loop via oxidized low-density lipoprotein (ox-LDL) deposition, foam-cell formation, and cytokine release. Endothelial metabolic dysregulation, including altered glycolysis and mitochondrial function, further exacerbates vascular injury and accelerates plaque development (Libby, 2021; Moore et al., 2013; Gimbrone and García-Cardeña, 2016).

Macrophages are the most important immune inflammatory cells in atherosclerotic lesions and play a core role at all stages of the disease process (Bashore et al., 2024; Murray and Wynn, 2011). Following endothelial injury, monocytes are recruited to the lesion site, differentiate into macrophages, and internalize excess lipoproteins to form cholesterol-rich foam cells (Libby, 2021; Moore et al., 2013). These macrophages shape the plaque immune microenvironment and exhibit a spectrum of activation states beyond the classical M1/M2 dichotomy. Single-cell studies have identified inflammatory, lipid-handling, proliferative, and smooth-muscle-like subsets, underscoring their phenotypic plasticity (Bashore et al., 2024). The canonical M1/M2 paradigm, originally derived from the Th1/Th2 framework, describes two extremes of macrophage activation: M1 macrophages, induced by Th1 cytokines, mediate inflammation and tissue injury, whereas M2 macrophages, driven by Th2 cytokines, secrete IL-10 and promote anti-inflammatory and reparative processes (Murray et al., 2014; Gordon, 2003). Dysregulated polarization, characterized by an imbalance in M1/M2 states, is a key mechanism driving AS progression (Makuch et al., 2024; Luo et al., 2024; Yang et al., 2020). Studies have shown that multiple signaling pathways, including TLR4/NF-κB/MAPK (Meng et al., 2023; Huang W. et al., 2024; Wang D. et al., 2023; Zheng Q. et al., 2025), PPAR-γ (Zahr et al., 2023; Bai et al., 2017; Zheng Y. et al., 2025), JAK/STAT (Yu et al., 2025; Wang J. et al., 2024; Chen J. et al., 2025), and PI3K/Akt (Liu et al., 2019; Zhang et al., 2021; Li P. et al., 2023; Fruman et al., 2017), regulate macrophage polarization and influencing the progression of AS. Consequently, targeting the dynamic balance of macrophage polarization through modulation of these signaling pathways represents a promising therapeutic strategy for AS.

Notably, Traditional Chinese Medicine (TCM)-derived bioactive metabolites and herbal formulas have emerged as unique modulators of macrophage plasticity, offering multi-pathway interventions with low toxicity (Zhi et al., 2023; Jian et al., 2019; Liu et al., 2020a; Liu et al., 2020b). We have integrated the available evidence to explore the regulatory mechanisms of key signaling pathways that influence macrophage phenotype during the plaque progression of AS. It is important to note that the vast majority of mechanistic insights and efficacy data discussed in this review are derived from robust preclinical models, including cell cultures and animal studies. These models are indispensable for elucidating complex biological pathways. Furthermore, this paper provides a comprehensive summary of the existing preclinical research and the preliminary clinical evidence regarding the prevention and treatment of AS using bioactive metabolites and herbal formulas. While several bioactive metabolites and herbal formulas have shown promise in early-stage clinical trials, the clinical evidence base remains less extensive than the preclinical foundation. This review comprehensively synthesizes evidence on the targeted modulation of AS-associated macrophage polarization by bioactive metabolites and herbal formulas, with a unique emphasis on the role of TCM as a multi-target regulator of macrophage plasticity. This approach provides novel perspectives for the prevention and treatment of AS.

## 2 Methods

This review utilized multiple literature search strategies. Authoritative databases were searched, including PubMed (http://www.ncbi.nlm.nih.gov/pubmed), Web of Science (https://www.webofscience.com), China National Knowledge Infrastructure (CNKI, https://www.cnki.net/), and Wanfang Data (http://www.wanfangdata.com.cn/). The search was conducted using a combination of subject terms and free-text words. Keywords included “AS,” “macrophage polarization,” “signaling pathways,” “bioactive metabolites,” and “herbal formulas.” This study only includes results discovered before May 2025. The search strategies were adapted to the characteristics of each database to ensure comprehensiveness and accuracy. Studies were included if they focused on AS and its molecular mechanisms related to macrophage polarization and therapeutic interventions involving bioactive metabolites or herbal formulas. Literature was excluded if it was irrelevant to the topic, lacked sufficient experimental design, contained incomplete data, or was not available in full text (The botanical drugs names were checked at http://mpns.kew.org/mpns-portal/).

## 3 Overview of AS and macrophage polarization

### 3.1 Formation of AS

**FIGURE 1:**
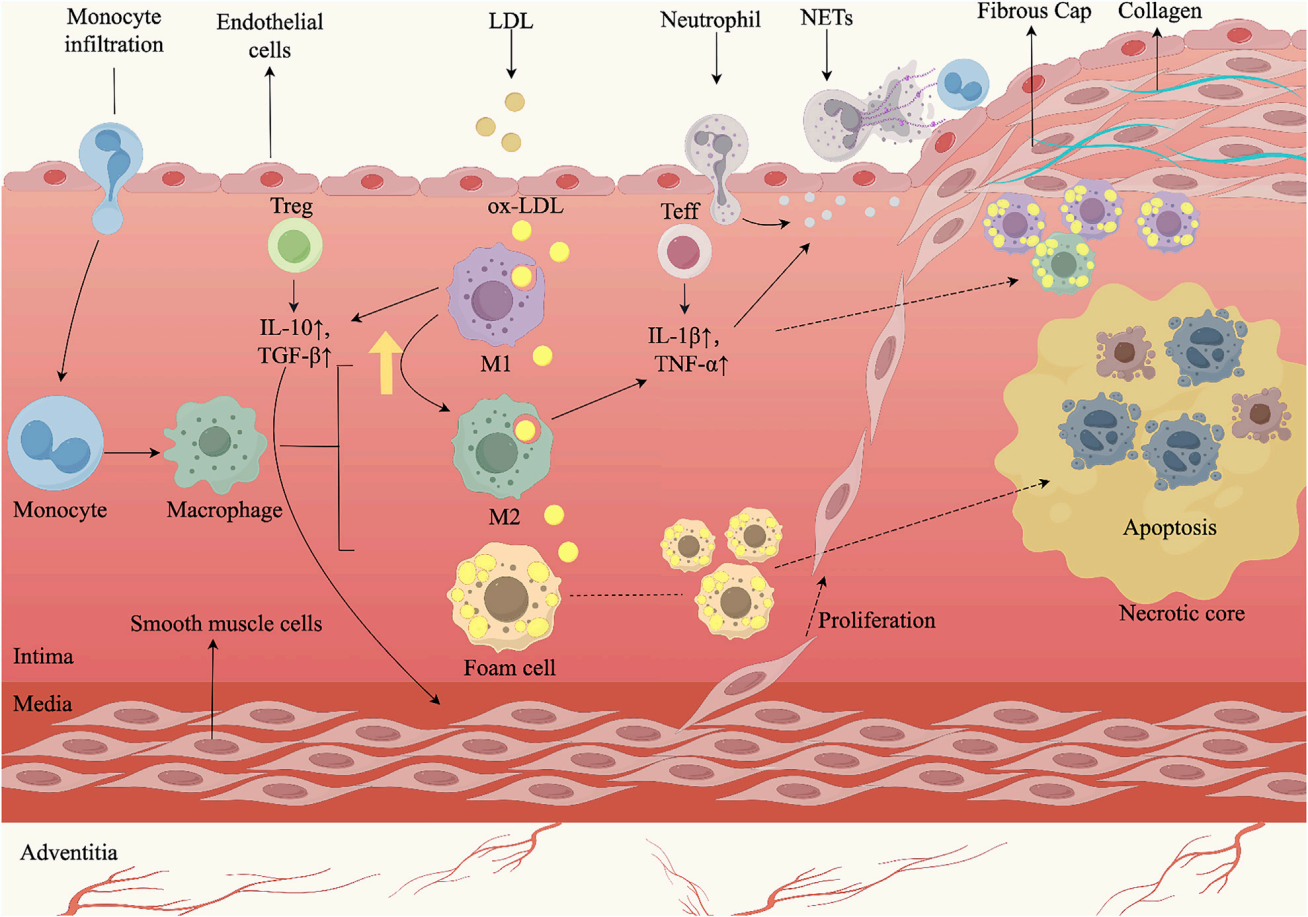
AS arises from multicellular interactions and complex signaling cascades within the arterial wall. Endothelial injury initiates the process by recruiting circulating monocytes, which adhere to activated endothelium, infiltrate into the intima, and differentiate into macrophages. Following uptake of oxLDL, macrophages transform into foam cells, whose accumulation seeds the lipid core and triggers early plaque growth. During lesion progression, macrophages and effector T cells (Teff) secrete pro-inflammatory cytokines such as IL-1β and TNF-α, which stimulate SMCs migration from the media, their proliferation, and subsequent thickening of the vessel wall. M1-polarized macrophages and Teff further amplify inflammatory signaling. By contrast, M2-polarized macrophages and regulatory T cells (Treg) secrete anti-inflammatory mediators including IL-10 and TGF-β, which suppress excessive inflammation and facilitate M1-to-M2 repolarization, thereby contributing to lesion stabilization. In advanced plaques, persistent SMC proliferation and extracellular matrix deposition form a fibrous cap over the necrotic core. However, neutrophil extracellular traps (NETs) exacerbate endothelial injury and promote cell death, destabilizing the plaque and predisposing to cap rupture. Rupture exposes thrombogenic material, triggering platelet aggregation and thrombus formation, which may result in acute cardiovascular events. The figure was created with Figdraw.com.

AS is a chronic, progressive inflammatory disease of the arterial wall, driven by immune dysregulation and lipid metabolism disorders (Roy et al., 2022; Engelen et al., 2022; Waksman et al., 2024). Its pathogenesis involves the activation of endothelial cells (ECs), monocytes, macrophages, smooth muscle cells (SMCs), and neutrophils (Doddapattar et al., 2022). Upon exposure to ox-LDL, ECs express chemokines and adhesion molecules, increasing vascular permeability and recruiting leukocytes (Bu et al., 2023). Monocytes infiltrate the intima, differentiate into macrophages or dendritic cells, and—together with SMCs—form foam cells that perpetuate inflammation (Liu W. et al., 2014). Foam cells release pro-inflammatory cytokines, damaging ECs and driving SMC proliferation, plaque growth, and restenosis (Liu X. et al., 2023). SMC-derived extracellular matrix (ECM) forms a fibrous cap over a necrotic, lipid-rich core undergoing calcification (Grootaert and Bennett, 2021). As plaque progresses, foam cells and SMCs undergo apoptosis or necrosis, leading to the accumulation of dead cells, cellular debris, and extracellular materials within the necrotic core (Gimbrone and García-Cardeña, 2016). Continued cell death and immune activation promote matrix metalloproteinases (MMPs)-mediated cap degradation, reducing plaque stability. Neutrophil-derived ECM-degrading enzymes further thin the cap, leading to rupture and thrombosis (Ovchinnikova et al., 2009). The mechanism by which multicellular interactions drive the development of AS plaques (Figure 1).

### 3.2 Macrophages in AS

Macrophages are key immune cells involved in the pathogenesis of AS. Following endothelial injury, circulating monocytes infiltrate the intima, differentiate into macrophages, and internalize excess apolipoprotein B–containing lipoproteins via scavenger receptors (e.g., SR-A1), generating foam cells that seed the lipid core (Wang B. et al., 2022; Chistiakov et al., 2017; van Tits et al., 2011). Foam-cell–driven inflammatory signaling and matrix remodeling promote smooth muscle cell proliferation and fibrous-cap weakening, reducing plaque stability (Liu Q. et al., 2022; Theofilis et al., 2023; Seifert et al., 2018). Progressive plaque accumulation narrows the lumen and predisposes to rupture, calcification, and thrombosis, causing ischemic injury (Kavurma et al., 2017; Moore and Tabas, 2011). This pathological milieu robustly promotes a shift towards the pro-inflammatory M1 phenotype, which is induced by signals like IFN-γ and LPS (Gordon, 2003). M1 macrophages secrete cytokines (e.g., TNF-α, IL-6) and matrix metalloproteinases (MMPs) that amplify inflammation at lesion sites and compromise fibrous cap integrity, thereby increasing plaque vulnerability (Li P. et al., 2021; Virga et al., 2021; Seifert et al., 2018). They are typically enriched in vulnerable plaque regions, and their apoptosis contributes to the necrotic core expansion (Hou et al., 2023). In contrast, the alternative M2 phenotype, induced by Th2 cytokines such as IL-4 and IL-13, secretes anti-inflammatory cytokines (e.g., IL-10) and profibrotic mediators (e.g., TGF-β) to promote tissue repair, enhance plaque stability, and facilitate lesion regression (Rao et al., 2022; Qiu et al., 2022; Locati et al., 2020). M2 macrophages are predominantly localized to more stable plaque areas (Hou et al., 2023). Local cues—including inflammation, cholesterol crystals, and oxidative stress—shape macrophage activation states beyond the classical M1/M2 dichotomy, revealing IL1^hi^ inflammatory, TREM2^+^ foam cell–like, proliferative, and ACTA2^+^ smooth-muscle–like subsets (Bashore et al., 2024; Li B. et al., 2022). The dynamic balance between M1 and M2 macrophages is a critical determinant of AS progression, and targeting macrophage polarization—by inhibiting M1 activation and promoting M2 differentiation—represents a promising therapeutic approach to suppress inflammation, limit necrotic core formation, and stabilize plaques (Wu J. et al., 2023; Wei et al., 2024; Blagov et al., 2023; Jinnouchi et al., 2020; Yu L. et al., 2023). In this context, TCM strategies aim to rebalance M1/M2 phenotypes and modulate foam cell–associated subsets, providing disease-modifying potential in AS.

## 4 Signaling pathways related to macrophage polarization in AS

During the progression of AS, the activation of multiple signaling pathways leads to dysregulated expression of inflammatory factors (Wu J. et al., 2023; Zhang et al., 2024). Key regulatory axes include Toll-like receptor 4 (TLR4)/nuclear factor-kappa B (NF-κB), peroxisome proliferator-activated receptor γ (PPAR-γ), Janus kinase (JAK)-signal transducer and activator of transcription (STAT), phosphoinositide 3-kinase (PI3K)/protein kinase B (Akt) pathway, and mitogen-activated protein kinase (MAPK) pathway, among others. These cascades drive discrete transcriptional programs that bias macrophages toward pro-inflammatory or pro-resolving states and thereby influence plaque vulnerability, necrotic-core expansion, and fibrous-cap integrity (Meng et al., 2022; Geiß et al., 2022). Accordingly, TCM act on these actionable nodes to re-tune macrophage programs in ways that are mechanistically aligned with AS control.

### 4.1 TLR4 signaling pathway

TLRs in macrophages recognize pathogen-associated molecular patterns (PAMPs), dimerize, and signal predominantly through MyD88 to activate the interleukin-1 receptor-associated kinase–tumor necrosis factor receptor-associated factor 6 (TRAF6)–IKK (IκB kinase) axis, converging on NF-κB and MAPK cascades (Litak et al., 2020). Cell-surface TLR1/2/4/5/6/10 are engaged by microbial ligands; among them, TLR4 is highly expressed across stages of atherosclerotic plaque formation and couples strongly to pro-inflammatory outputs (Jin M. et al., 2023; Bagheri et al., 2024). Functionally, prototypical agonist LPS activates TLR4–MyD88 signaling to enhance NF-κB p65 phosphorylation and MAPKs, promoting M1 polarisation with increased TNF-α, IL-6, and iNOS; conversely, genetic or pharmacologic reduction of TLR4 favors M2 traits and improves inflammatory conditions such as AS (Yan et al., 2024; Wu et al., 2024; Liu F. et al., 2024; Rumpel et al., 2024; Sun et al., 2022). These mechanisms highlight TLRs as potential therapeutic targets. In the context of TCM, metabolites like Alkaloids and Polyphenols have been shown to inhibit TLR4/NF-κB signaling (Li et al., 2020; Zhou et al., 2015), indicating their potential to modulate macrophage phenotype toward M2, which may contribute to the management of cardiovascular diseases including AS.

### 4.2 NF-κB signaling pathway

NF-κB is a conserved transcription factor family (p50/p105, p52/p100, RelA/p65, c-Rel, RelB) that is activated predominantly via IKK-dependent phosphorylation and degradation of IκB, enabling p65/p50 nuclear translocation to initiate inflammatory gene programs (Florio et al., 2022). This pathway is autoregulated by NF-κB–driven IκB resynthesis, which limits excessive nuclear residency and maintains immune homeostasis (An et al., 2021). Sustained NF-κB activation upregulates TNF-α, IL-6, and iNOS and drives macrophage polarisation toward the M1 phenotype, whereas attenuation of this axis favors M2-associated anti-inflammatory traits; aberrant stimuli can also reprogram M2 cells back to M1 via NF-κB (Li J. et al., 2023; Huang YM. et al., 2024; Geiß et al., 2022). This is particularly relevant for TCM, where numerous bioactive metabolites and herbal formulas have been reported in preclinical studies to suppress p65 phosphorylation, impede nuclear translocation, or stabilize IκB, thereby curbing M1 polarization bias and highlighting a strategic avenue for TCM to recalibrate macrophage responses and restore immune balance, with implications for AS and related inflammatory pathologies (Chen and Chen, 2019; Fan et al., 2022).

### 4.3 MAPK signaling pathway

The MAPK pathway, comprising extracellular signal-regulated kinase (ERK), c-Jun N-terminal kinase (JNK) and p38 modules, is engaged by receptor and stress cues that funnel through a three-tiered kinase cascade (MAPKKK→MAPKK→MAPK) to reprogram transcription and stress responses in vascular cells (Achkar et al., 2018; Binion et al., 2009). Once activated, MAPKs phosphorylate selective substrates and transcription factors, coordinating proliferation–survival decisions and inflammatory gene expression that shape vascular lesion biology (Tang et al., 2022; El-Sayed et al., 2021). Activation of the p38 and JNK pathways is particularly instrumental in driving macrophages toward the pro-inflammatory M1 phenotype by upregulating cytokines like TNF-α and IL-1β, a process evident in AS (Shen et al., 2023). Conversely, certain signals through the ERK pathway can promote the anti-inflammatory M2 phenotype, highlighting the complex role of MAPKs in maintaining polarization balance (Wang Y. et al., 2023). Therefore, targeted modulation of specific MAPK branches represents a viable strategy for treating inflammatory diseases by reprogramming macrophage polarization. Glycosides from TCM have demonstrated efficacy in attenuating inflammation by selectively inhibiting p38 or JNK signaling, approach for the treatment and intervention of inflammatory diseases such as AS (He et al., 2023).

### 4.4 PPAR-γ signaling pathway

PPAR-γ is a ligand-activated nuclear receptor that integrates lipid/energy metabolism with inflammation control; in macrophages, its activation coordinates cholesterol efflux programs (e.g., ABCA1/ABCG1) and transrepresses pro-inflammatory transcription factors (NF-κB, AP-1, STAT), thereby coupling metabolic homeostasis to immune tone (Chinetti et al., 2003; Pascual et al., 2005; Szanto et al., 2010). AMP-activated protein kinase (AMPK) functions upstream to potentiate PPAR-γ activity and metabolic rewiring, positioning the AMPK→PPAR-γ axis as a nexus between cellular energy status and macrophage gene programs (Cui et al., 2023; Zhou et al., 2023). Functionally, PPAR-γ activation shifts macrophages from an M1 to an M2 phenotype, dampens pro-inflammatory cytokines, and improves the lesional milieu, whereas reduced activity favors inflammatory skewing (Abdalla et al., 2020; Bouhlel et al., 2007). Herbal formula are being explored to modulate the AMPK→PPAR-γ axis (e.g., enhancing AMPK activity, limiting p65 nuclear translocation, or reinforcing cholesterol efflux), which can promote a shift toward the M2 phenotype, underscoring the value of TCM in targeting metabolic-inflammatory crosstalk to mitigate disease progression.

### 4.5 JAK/STAT signaling pathway

The JAK/STAT pathway couples extracellular cytokine–receptor engagement to transcriptional reprogramming: Upon binding of cytokines to their cognate receptors, receptor-associated JAK kinases are activated and phosphorylate STAT transcription factors (Bi et al., 2019). The phosphorylated STATs then dimerize and translocate to the nucleus to regulate the expression of genes defining macrophage functional phenotypes (Xin et al., 2020; Sarapultsev et al., 2023). Critically, the specific STAT protein activated determines the polarization outcome: STAT1 activation by IFN-γ drives a robust pro-inflammatory M1 phenotype, whereas IL-4/IL-13 signaling via STAT6 induces an anti-inflammatory M2 phenotype (Gong et al., 2017; Zheng et al., 2024; Ding et al., 2024; Li ZH. et al., 2023). The balance between these opposing signals is a key determinant of plaque inflammation and stability. Given this pivotal role, the JAK-STAT pathway represents a promising therapeutic target for modulating macrophage polarization in AS. By tempering STAT1 signaling and/or amplifying STAT6-driven programs, TCM reweight JAK–STAT outputs toward anti-inflammatory macrophage identities, which provides a plausible molecular foundation for the ability of TCM interventions to suppress pro-inflammatory M1 polarization and promote resolution of inflammation in AS.

### 4.6 PI3K/Akt signaling pathway

The PI3K/Akt pathway integrates receptor and TLR inputs to lipid signaling: class I PI3K generates PIP3, which recruits Akt to the membrane for activation by PDK1 and mTORC2, thereby coordinating metabolism, survival, and inflammatory gene control (Monaci et al., 2021; Abeyrathna and Su, 2015). Akt activation generally promotes an anti-inflammatory M2 phenotype, particularly through the downstream mTORC1 axis, which supports M2-associated metabolic reprogramming (Rocher and Singla, 2013). However, the pathway can also contribute to M1 polarization under specific conditions, for instance, by cross-talking with and enhancing NF-κB signaling (Babaev et al., 2016). The distinct roles of Akt isoforms (e.g., Akt1 vs. Akt2) further add a layer of complexity to this regulation (Arranz et al., 2012). Selected TCM-related products fine-tune PI3K–Akt–mTOR activity—for example, by blunting maladaptive Akt phosphorylation or reshaping metabolic flux—thereby illustrating how TCM can precisely influence immune-metabolic pathways to favor anti-inflammatory macrophage polarization, highlighting their therapeutic potential.

### 4.7 NRF2 signaling pathway

Nuclear factor erythroid 2–related factor 2 (NRF2) is a redox-sensitive transcription factor that, upon release from Keap1, translocates to the nucleus to bind antioxidant response elements and induce cytoprotective programs (e.g., glutathione synthesis, HO-1), thereby lowering oxidative stress and coupling redox control to lipid handling and foam-cell biology (Meng et al., 2022; Ooi et al., 2017; Wu et al., 2022). Hemodynamic cues (laminar shear), endothelial inflammation, and lipid peroxidation activate NRF2 in vascular beds, linking redox homeostasis to atheroprotection and lesion remodeling (Hosoya et al., 2005; He L. et al., 2024). In foam cells, NRF2 helps balance cholesterol uptake and efflux, integrating oxidative and metabolic signals during atherogenesis (Wu et al., 2022). NRF2 restrains M1 polarisation by repressing pro-inflammatory mediators (IL-6, IL-1β, TNF-α) and can facilitate reparative/M2 traits; enhancing NRF2 stability via U-box containing protein 1 silencing further promotes M2 polarisation and reduces oxidative injury (Kobayashi et al., 2016; Mimura and Itoh, 2015; Li et al., 2025). Context-dependence exists: in certain settings NRF2 activation may favor CD163^+^ subsets and CD36-mediated lipid uptake, potentially accelerating lesion growth (Liu J. et al., 2020). Overall, evidence supports NRF2’s dominant antioxidant/anti-inflammatory role—NRF2 deficiency aggravates plaque inflammation, calcification, and fibrous-cap thinning—underscoring its importance for plaque stability (Alonso-Piñeiro et al., 2021; Bozaykut et al., 2014; Ruotsalainen et al., 2019). TCM-derived metabolites such as oridonin (activates NRF2 and inhibits NOD-like receptor protein 3 (NLRP3), reducing macrophage infiltration/oxidative stress and stabilising plaques in ApoE^−/−^ mice) and quercetin (binds Keap1 Arg483 to activate NRF2, lower oxidative stress, and suppress macrophage pyroptosis) exemplify tractable NRF2-targeting strategies (Wang L. et al., 2023; Luo et al., 2022); broader classes including flavonoids and terpenoids show similar NRF2-mediated anti-inflammatory/antioxidant effects. A key open question is whether specific metabolites can selectively modulate NRF2 to direct macrophage polarisation, warranting systematic investigation for next-generation AS therapeutics.

## 5 Research on the intervention of AS through the regulation of macrophage polarization-related pathways by TCM

According to ancient Chinese medical records and modern research, TCM has unique advantages in treating diseases through multi-target, multi-pathway mechanisms with minimal toxicity and side effects. In various pathological stages, from endothelial dysfunction to plaque rupture, TCM regulates macrophage polarization to inhibit excessive inflammatory responses, enhance plaque stability, and thus delay the progression of AS (Wang W. et al., 2024; Jian et al., 2019).

**FIGURE 2:**
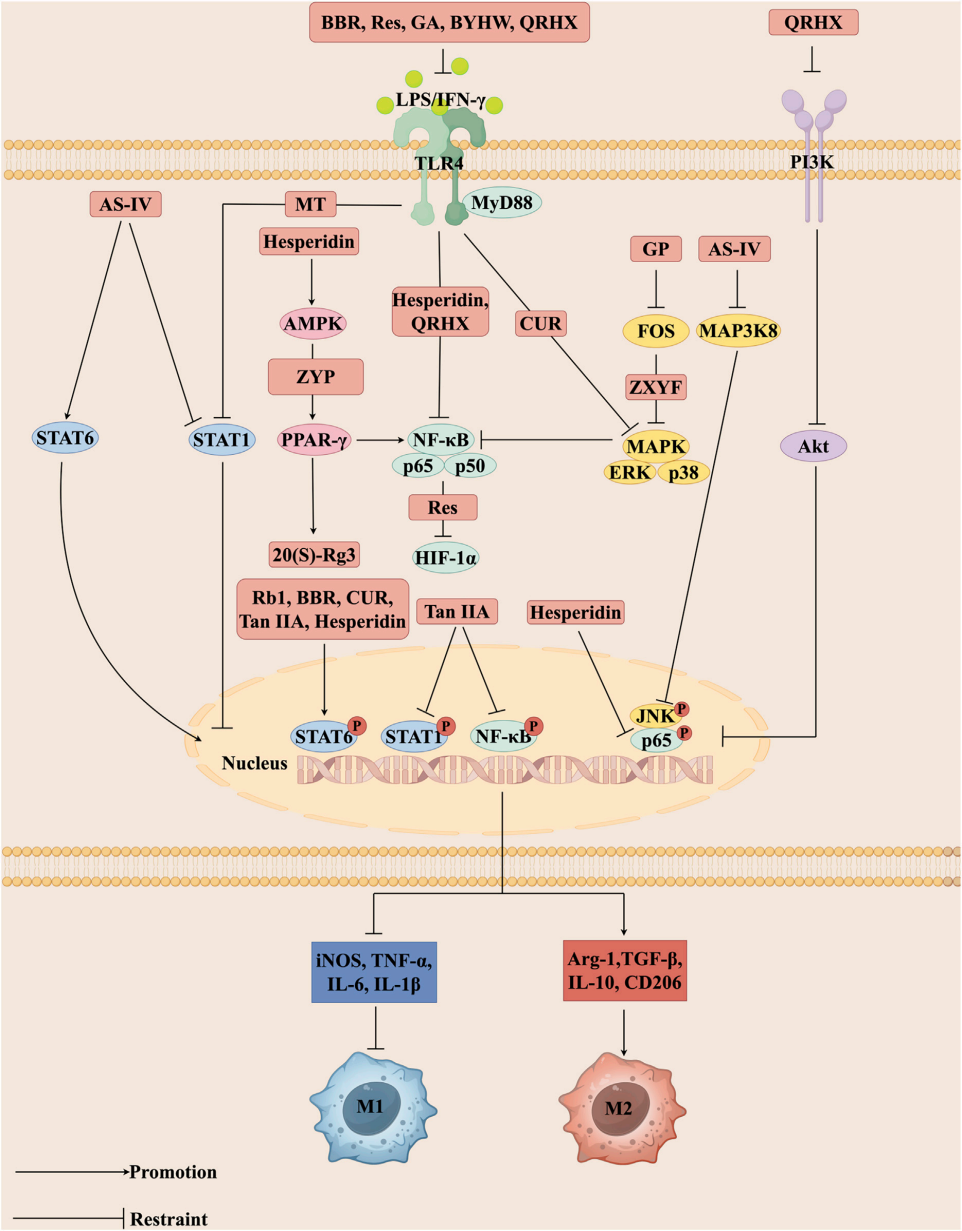
Different bioactive metabolites, including Rb1, Rg3, BBR, CUR, MT, Tan IIA, Res, GA, GP, AS-IV, and Hesperidin as well as herbal formulas such as BYHW, ZYP, ZXYF, and QRHX regulate signaling pathways central to macrophage polarization. These interventions act on key nodes such as TLR4, MAPK, PPAR-γ, and JAK/STAT, thereby modulating intracellular phosphorylation events, including STAT6 and NF-κB nuclear translocation. Through these mechanisms, they alter the expression of inflammatory mediators, promote the polarization of macrophages toward the anti-inflammatory M2 phenotype, and suppress pro-inflammatory M1 activity. Collectively, this rebalancing of macrophage function contributes to the attenuation of vascular inflammation, stabilization of atherosclerotic plaques, and overall improvement in the pathological process of atherosclerosis (AS). The figure was created with Figdraw.com.

The use of medicinal plants continues as an alternative treatment for various diseases, including cardiovascular disease (Shaito et al., 2020). With the progress of modern society and advances in medical research, various botanical drugs have demonstrated potential application value in the treatment of AS. Bioactive metabolites, isolated from botanical drugs, are structurally defined small molecules with specific biological activities, serving as crucial sources for drug discovery (such as alkaloids, flavonoids, terpenoids, polyphenols, and glycosides) (Pawlita et al., 1985; Cheng et al., 2017). The application value of TCM in the prevention and treatment of AS has been supported by modern pharmacological research. Increasingly, bioactive metabolites and herbal formulas are reported to delay AS, providing a scientific basis for the development of related drugs. This article systematically summarizes bioactive metabolites and herbal formulas regulate macrophage polarization in the treatment of AS. The intervention mechanisms of bioactive metabolites and herbal formulas on macrophage polarization-related signaling pathways (Figure 2).

### 5.1 Bioactive metabolites

#### 5.1.1 Ginsenoside

Ginseng refers to the dried root of the species *Panax ginseng* C.A.Mey. of the *Araliaceae* family (Liu H et al., 2020), which has both medicinal and dietary value (Jang et al., 2023). Ginsenosides, a class of triterpene metabolites found in P. *ginsen*g, have demonstrated therapeutic effects in atherosclerotic disease. For example, ginsenosides delay AS progression by regulating inflammation-related signaling pathways and lipid metabolism disorders (Sun et al., 2016). To date, ginsenosides extracted from ginseng are classified into two main categories: pentacyclic triterpenes (pentacyclic oleanolic acid type) and tetracyclic triterpenes (tetracyclic dammarane type) (Liang et al., 2024). These structural categories underpin the diverse pharmacological activities of ginsenosides, particularly their immunomodulatory and lipid-regulating effects in AS. Ginsenoside Rb1 (Rb1) is the most abundant bioactive metabolites in ginseng and has multiple effects in preventing and treating AS, including anti-inflammatory, antioxidant, improving myocardial ischemia, and anti-angiogenesis properties (Zhou et al., 2018; Ni et al., 2022). In a study utilizing ApoE^−/−^ mice and primary peritoneal macrophages isolated from C57BL/6 mice, Zhang X. et al. (2018) reported that Rb1 promotes the secretion of IL-4 and IL-13 in peritoneal macrophages. They further showed that Rb1 dose-dependently enhances phosphorylation of STAT6, thereby facilitating a shift in macrophage phenotype toward the anti-inflammatory M2 state. In addition, Rb1 increases IL-10 expression, decreases MMP-9 levels, mitigates AS-associated inflammatory responses, and contributes to enhanced plaque integrity. Rg3, a natural ligand of PPAR-γ, has been shown to specifically bind to this receptor (Kwok et al., 2012). Thus, Rg3 targets PPAR-γ to remodel macrophage polarization from the pro-inflammatory M1 to the anti-inflammatory M2 phenotype, thereby inhibiting AS progression (Kwok et al., 2012). Guo et al. (2018) identified the PPAR-γ signaling pathway as a key mediator of the anti-AS actions of 20(S)-Rg3. *In vitro*, using bone marrow-derived macrophages (BMDMs), RAW264.7, and THP-1 cells, 20(S)-Rg3 activates PPAR-γ to drive a phenotypic switch in macrophages from M1 to M2, thereby suppressing pro-inflammatory mediators (e.g., iNOS, IL-6 and TNF-α) while enhancing anti-inflammatory factors (e.g., Arg-1, IL-10 and TGF-β). This immunomodulatory effect alleviates advanced glycation end-products (AGEs)-induced inflammation and improves the vascular microenvironment. Moreover, in ApoE^−/−^ mice, Rg3 enhances plaque structural integrity by reducing lipid deposition, promoting smooth muscle cell proliferation, and increasing collagen expression (Xue et al., 2021). In diabetic ApoE^−/−^ mice, Rg3 suppresses pro-inflammatory M1 polarization while fostering anti-inflammatory M2 activation. Notably, these effects are reversed upon co-administration of a PPAR-γ antagonist, which exacerbates inflammatory responses. Consistent with these *in vitro* findings, studies in diabetic ApoE^−/−^ mice demonstrated that 20(S)-Rg3 reduces lipid accumulation, increases collagen deposition, stabilizes atherosclerotic plaques, and lowers blood glucose levels, ultimately providing integrated protection against atherosclerotic progression through PPAR-γ activation (Guo et al., 2018). Cumulatively, these preclinical findings demonstrate that ginsenosides, especially Rb1 and Rg3, exert strong anti-inflammatory and plaque-stabilizing effects via STAT6 and PPAR-γ signaling. Nevertheless, the translation of these mechanistic insights into clinical benefit remains unproven. Current evidence is largely confined to cell and animal models, while high-quality clinical trials are scarce. Considering the complexity of AS and its clinical consequences, future research should move beyond mechanistic exploration toward rigorous evaluation of clinical endpoints, such as cardiovascular event reduction, long-term plaque stabilization, and metabolic improvement. Only by establishing robust translational frameworks can ginsenosides advance from promising experimental agents to evidence-based therapeutic options.

#### 5.1.2 Berberine

Berberine (BBR) is an isoquinoline present in Tinospora cordifolia and roots, rhizomes, and stem bark of several medicinal plants belonging to the Ranunculaceae, Rutaceae, and Berberidaceae families (Ryuk et al., 2012; Gawel et al., 2020). BBR exerts vascular protective effects, including anti-inflammatory, antioxidant, and lipid metabolism-regulating properties (Wu M. et al., 2020; Wu et al., 2021; Zhu et al., 2022). In addition, the broad pharmacological activities of BBR have been reported to act on multiple diseases, including diabetes, obesity, neurodegenerative, and neuropsychiatric disorders (Gasmi et al., 2024). It modulates multiple signaling pathways, such as STAT, MAPK, NF-κB, PI3K/Akt, and AMPK, to inhibit inflammatory cell infiltration, improve endothelial dysfunction, and enhance vascular remodeling, thereby preventing AS progression (Ai et al., 2021). Notably, the TLR4/MyD88/NF-κB axis is a key pathway through which BBR regulates inflammatory responses and vascular remodeling in AS (den et al., 2010). These mechanistic insights indicate that BBR confers multi-targeted vascular protection, mainly through regulation of inflammation, lipid metabolism, mitochondrial function, and the functional plasticity of macrophages. *In vitro* studies, such as those using RAW264.7 macrophages, demonstrate that BBR can alter the inflammatory profile of these cells by inhibiting the TLR4/MyD88/NF-κB pathway. This leads to reduced expression of M1-associated markers (e.g., iNOS, TNF-α, and IL-6) and elevated levels of M2-associated markers like CD206 (Li et al., 2020). Furthermore, BBR enhances phosphorylation and activation of the STAT6 signaling pathway, which contributes to its capacity to reprogram macrophage phenotype away from the pro-inflammatory M1 state and toward the anti-inflammatory M2 state (Chen YS. et al., 2024). In AS lesions, mitochondrial dysfunction elevates ROS levels, which can promote a pro-inflammatory macrophage phenotype and compromise plaque structural integrity. BBR helps preserve mitochondrial function, thereby mitigating oxidative stress and contributing to a more stable plaque phenotype (Peng et al., 2019; Tan et al., 2020). These preclinical studies support the anti-inflammatory, lipid-lowering, and plaque-stabilizing roles of BBR in AS models. Nevertheless, most available evidence is derived from experimental settings, and critical questions regarding its pharmacokinetics, optimal dosing, long-term safety, and interactions with standard-of-care therapies remain unanswered. To advance BBR toward clinical utility, future research should not only confirm its vascular protective effects in human subjects but also integrate evaluations of hard clinical endpoints, such as cardiovascular morbidity and mortality. Addressing these translational gaps will be essential to determine whether BBR can evolve from a multi-target experimental agent into a viable therapeutic candidate in the management of atherosclerotic disease.

#### 5.1.3 Curcumin

Curcumin (CUR) is a natural polyphenolic diarylheptanoid extracted from the rhizomes of species belonging to the Zingiberaceae and turmeric families, especially *Curcuma longa* L (Lv et al., 2020). Extensive preclinical studies in animal models have demonstrated its multi-targeted pharmacological activities, including anti-inflammatory, antithrombotic, antiviral, anticancer, anti-degenerative disease, hepatoprotective, and neuroprotective effects, which are largely mediated through multiple molecular targets (Laurindo et al., 2023; Corrêa Carvalho et al., 2024; Sharifi-Rad et al., 2020). At the mechanistic level, CUR exerts anti-inflammatory effects through multiple pathways, including inhibition of TLR4 overexpression and dimerization, suppression of MAPK pathway phosphorylation, prevention of IκBα degradation, and impairment of nuclear translocation of the NF-κB subunit p65 (Lubbad et al., 2009). Particularly, its interference with the NF-κB pathway—a central regulator of inflammation—extends to attenuating macrophage infiltration within atherosclerotic plaques (Zhang S. et al., 2018). Moreover, CUR modulates macrophage phenotype by directly stabilizing IκBα, thereby restraining the pro-inflammatory M1 state, and concurrently activating PPAR-γ to favor the anti-inflammatory M2 phenotype (Chen and Xu, 2014). These multi-pathway mechanisms suggest that CUR may regulate inflammation and immune balance in AS by modulating macrophage polarization and inflammatory signaling. In cellular models, such as THP-1-derived macrophages, CUR dose-dependently suppresses the production of pro-inflammatory cytokines including TNF-α, IL-6, and IL-12B (p40). This anti-inflammatory effect is linked to inhibition of the TLR4/MAPK/NF-κB axis, which collectively drives a phenotypic shift in macrophages from a pro-inflammatory M1 toward an anti-inflammatory state (Zhou et al., 2015). These findings underscore the potential of CUR as a promising therapeutic agent for AS (Zhou et al., 2015). Moreover, combined treatment with CUR and BBR in RAW264.7 macrophages ameliorates the pathological progression of AS by enhancing STAT6 phosphorylation and suppressing the expression of M1 macrophage markers (Chen YS. et al., 2024). Integrating these preclinical data reveals that CUR not only reduces inflammatory mediator expression but also favors an M2-like functional state in macrophages. Moreover, it exhibits synergistic effects with other natural metabolites such as berberine to exert anti-atherosclerotic actions. Clinical studies have indicated that CUR supplementation may improve surrogate markers, including systemic inflammation and oxidative stress; however, the evidence is largely confined to intermediate endpoints and short-term interventions. Future investigations should therefore move beyond biomarker-based observations and establish whether CUR can achieve durable benefits on hard cardiovascular outcomes, such as plaque stabilization, event reduction, and long-term vascular protection. In addition, issues related to bioavailability, optimal formulation, and integration into standard therapeutic regimens require careful evaluation to clarify its true translational potential in the management of AS.

#### 5.1.4 Matrine

Matrine (MT), also known as oxymatrine or Sophora alkaloid, is an alkaloid obtained from different species of the *Sophora* genus (Wang X. et al., 2023; Zhang M. et al., 2023). MT plays a crucial role in several pathophysiological processes, including antioxidant, anti-ischemia–reperfusion injury (Lu Q. et al., 2021), anti-sepsis (Wang X. et al., 2023), antiviral (Qiao et al., 2024), intestinal barrier protective (Yu D. et al., 2023), antidepressant (Zhang M. et al., 2023), analgesic, and neuroprotective (Zhu C. et al., 2024) effects. Mechanistically, MT interferes with a network of signaling cascades that drive AS progression. Specifically, MT inhibits the TGF-β1/Smad pathway, leading to suppression of ECM formation and reduced expression of inflammatory factors (Chen C et al., 2024). In addition, in ox-LDL-stimulated vascular smooth muscle cells, MT suppresses the activation of NF-κB, MAPK, and JAK/STAT3 signaling pathways, resulting in the downregulation of pro-inflammatory mediators such as IL-1β, TNF-α, VCAM-1, and ICAM-1, thereby attenuating inflammatory responses and cellular adhesion (Lu et al., 2018). These findings suggest that MT exerts vascular protection by targeting inflammation, oxidative stress, and extracellular matrix remodeling. Treatment of M0-polarized primary mouse peritoneal macrophages with MT effectively inhibited AGEs-induced M1 macrophage polarization through suppression of the TLR4/STAT1 signaling pathway (Cui et al., 2022). Concurrently, MT was found to downregulate the expression of DNA methyltransferases in macrophages, an effect associated with mitigated oxidative stress and delayed AS development (Cui et al., 2022). Additionally, studies have demonstrated that MT ameliorates inflammation and attenuates vascular wall thickening in high-fat diet (HFD)-fed mice (Liu et al., 2016). Collectively, these preclinical observations delineate a role for MT in counteracting AS by modulating macrophage function, restraining pro-inflammatory signaling, and limiting pathological vascular remodeling. However, current evidence remains almost exclusively experimental, and the clinical landscape for MT in AS is largely unexplored. Given its extensive pharmacological spectrum and long history of use in traditional medicine, future research should focus on bridging mechanistic insights with patient-oriented outcomes. Particular attention is needed to clarify its pharmacokinetics, optimal therapeutic window, and long-term safety profile in the context of cardiovascular disease. Establishing whether MT can translate its multi-target anti-atherosclerotic effects into measurable improvements in cardiovascular events will be pivotal for advancing its development as a viable therapeutic candidate.

#### 5.1.5 Resveratrol

Resveratrol (Res) is a natural polyphenolic compound, which is present in a variety of plants and their products, such as *Polygonum cuspidatum*, grape seeds, peanut and so on (Ma et al., 2023). Res possesses anti-inflammatory, antioxidant, lipid-regulating, and cardioprotective effects, making it effective against chronic inflammatory diseases, including cardiovascular disease (CVD) (Meng et al., 2021; Zhang et al., 2021). These multiple signaling pathways highlight Res as a pleiotropic regulator of vascular inflammation and lipid metabolism in the context of AS. Its ability to broadly suppress pro-inflammatory signaling, as evidenced in both human monocyte-derived M1 and M2 macrophages challenged with 7-oxocholesterol, underscores its potential as a therapeutic agent for AS (Buttari et al., 2014). Guo et al. (2023) demonstrated that Res treatment in LPS+IFN-γ-induced RAW264.7 macrophages suppressed gene and protein expression in the TLR4/NF-κB/HIF-1α pathway. Consequently, Res dose-dependently restricted the pro-inflammatory M1 phenotype, contributing to its anti-AS properties. Furthermore, Res inhibits the degradation of IκB-α and the nuclear translocation of NF-κB p65 induced by TNF-α, thereby reducing arterial macrophage infiltration (Repossi et al., 2020; Shang et al., 2019). Moreover, Res contributes to enhanced plaque integrity and retards AS development. It also reduces ischemia-reperfusion injury and prevents AS-related vascular events (Raj et al., 2021). In addition, Res decreases AS-related markers such as MMP-9 and CD40 ligand in lesion areas, mitigating AS pathology (Ji et al., 2022). Together, these preclinical data indicate that Res orchestrates a multi-faceted anti-atherosclerotic response by tempering pro-inflammatory macrophage activation, dampening key inflammatory cascades, and reinforcing plaque structure, thereby slowing the progression of AS. Nonetheless, most studies remain confined to mechanistic and surrogate outcomes, and whether these effects can be translated into durable cardiovascular protection in humans is still unclear. Given the pleiotropic actions of Res, future investigations should not only validate its impact on clinically relevant endpoints,but also resolve critical challenges including bioavailability, inter-individual variability, and formulation strategies. Addressing these gaps will be essential to determine whether Res can progress from a promising experimental metabolites to a clinically actionable intervention in atherosclerotic disease.

#### 5.1.6 Tanshinone IIA

*Salvia miltiorrhiza* (Danshen), the dried rhizomes and roots of *Salvia miltiorrhiza* Bge (Lamiaceae), contains over 200 identified metabolites, including lipophilic diterpene quinones and water-soluble phenolic acids (Petitjean et al., 2022; Guo et al., 2020). Tanshinone IIA (Tan IIA), the most studied diterpene quinone, exhibits anti-inflammatory, anti-thrombotic, anti-apoptotic, and endothelial-protective effects in preclinical models of AS (Wang H. et al., 2020; Yang et al., 2023). Its metabolites, particularly Tan IIA, have also been investigated in studies targeting AS, hepatic steatosis, and diabetic nephropathy (Wu Q. et al., 2023). Specifically, the underlying mechanism is the metabolites of Salvia miltiorrhiza, especially Tan IIA, exert cardiovascular protection through multi-target actions, particularly by regulating inflammation, immune cell function, and plaque stability. Chen et al. (2019) demonstrated in ApoE^−/−^ mice that Tan IIA orchestrates macrophage phenotypic balance by concurrently modulating the STAT6 and NF-κB pathways. It upregulates phosphorylated STAT6, fostering the expression of M2-associated markers (TGF-β, Arg-1, IL-10), while simultaneously inhibiting NF-κB and STAT1 activation to suppress key M1-related mediators (TNF-α, iNOS, IL-12, IL-6), collectively favoring an anti-inflammatory macrophage phenotype and mitigating AS. Furthermore, in ApoE^−/−^ mice, Tan IIA modulates immune cell function and activation, reduces inflammatory factor levels, and restores abnormal signaling pathways (Guo et al., 2020). For example, Tan IIA inhibits the TLR4/MyD88/NF-κB pathway, reducing inflammation in a dose-dependent manner, decreasing macrophage infiltration, increasing collagen content, and stabilizing AS plaques (Chen Z. et al., 2019). Additionally, Tan IIA suppresses NF-κB activation, downregulates PPAR-γ protein expression, and reduces mRNA levels of IL-10, IL-6, and MMP-1, thereby blocking the propagation of inflammatory signals, stabilizing atherosclerotic plaques, and ultimately lowering the risk of plaque rupture (Chen and Xu, 2014). Wang N et al. (2020) further showed that combined treatment of Tan IIA and astragaloside IV (AS-IV) activates PI3K/Akt and inhibits TLR4/NF-κB signaling, significantly reducing IL-6, MMP-9, TNF-α, and C-reactive protein levels, while upregulating endothelial nitric oxide synthase. This combination reduced lipid deposition and stabilized plaques in ApoE^−/−^ mice. In conclusion, these collective preclinical findings position Tan IIA as a multi-faceted agent capable of dampening vascular inflammation, reprogramming macrophage responses, and enhancing plaque structural resilience in AS models. Yet, most available findings remain confined to cellular and animal experiments, and the translation of these benefits into clinical efficacy is far from established. While Tan IIA has demonstrated robust effects on immunomodulation and vascular protection, decisive evidence regarding its ability to alter the natural history of AS in humans is lacking. Future studies should extend beyond mechanistic observations to evaluate clinically relevant outcomes, including plaque regression, stabilization against rupture, and reduction in cardiovascular events. Moreover, careful assessment of pharmacokinetics, dosing strategies, and long-term safety will be essential for determining whether Tan IIA can advance from an experimental metabolites to a clinically applicable therapy in atherosclerotic disease.

#### 5.1.7 Ganoderic acid

*Ganoderma lucidum* (G. lucidum)is a medicinal mushroom with a long history of use for its tonic and health-promoting properties (Liu C. et al., 2023). G. lucidum is rich in various bioactive metabolites, including polysaccharides, triterpenoids, sterols, and other phytochemicals (Yuan et al., 2022). Ganoderic acid (GA), a triterpenoid metabolite extracted from G. lucidum, exhibits pharmacological activities such as anti-lipid accumulation (Li Y. et al., 2021; Zheng et al., 2023), anticancer (Chen S. et al., 2024), anti-aging (Chen L. et al., 2025), and anti-asthmatic effects (Lu X. et al., 2021). These diverse biological functions suggest that GA may act on multiple signaling pathways to regulate inflammation, lipid metabolism, and vascular homeostasis in the context of AS. Integrated *in vivo* and *in vitro* studies (ApoE^−/−^ mice, BMDMs, RAW264.7 cells) have shown that GA targets the TLR4/MyD88/NF-κB axis. This inhibition leads to reduced secretion of key pro-inflammatory cytokines (TNF-α, IL-6, IL-1β, MCP-1) and restrains pro-inflammatory macrophage activation. Consequently, GA administration dose-dependently diminishes the plaque necrotic core, augments collagen deposition, and improves plaque structural integrity (Quan et al., 2024). Additionally, GA regulates the release of inflammatory mediators by modulating key signaling pathways, including PI3K/Akt/mTOR, NF-κB, Neurogenic locus notch homolog protein 1, and JAK3/STAT3, which in turn influences the progression of AS (Wang S. et al., 2024). In general, the preclinical evidence underscores GA’s potential to mitigate AS through concerted anti-inflammatory actions, modulation of macrophage phenotype, and reinforcement of plaque stability. However, clinical investigations remain virtually absent, and the therapeutic significance of GA for human AS is uncertain. Given its long-standing use in traditional medicine and promising mechanistic profile, future work should aim to bridge preclinical efficacy with patient-centered outcomes. Particular emphasis should be placed on evaluating its ability to influence clinically relevant endpoints—such as plaque vulnerability, vascular function, and cardiovascular event rates—while also addressing challenges related to standardization of extracts, pharmacokinetics, and long-term safety. Establishing such evidence will be critical for defining the translational value of GA as a potential adjunctive therapy in atherosclerotic disease.

#### 5.1.8 Geniposide

*Gardenia jasminoides Ellis* (G. jasminoides) is an evergreen shrub of species belonging to the Rubiaceae family that grows widely in many regions of the world (Shen et al., 2020). Geniposide (GP), the major bioactive metabolite isolated from G. jasminoides, is a cycloartenol glycoside with anti-inflammatory, lipid-regulating, macrophage-modulating, endothelial-protective, and anti-thrombotic properties, which help prevent and treat AS, diabetes, and related complications (Li H. et al., 2022; Li D. et al., 2024). Several studies have demonstrated that GP regulates inflammatory mediator release by modulating key signaling pathways, including MAPK, PI3K, Akt, NF-κB, and TLR, thereby promoting M2 polarization (Chi et al., 2006; Ma et al., 2024). These pleiotropic actions indicate that GP exerts its protective role in AS through simultaneous regulation of inflammation, lipid metabolism, and vascular function. In a study on New Zealand white rabbits with HFD-induced AS, Jin et al. (Jin et al., 2020) demonstrated that GP downregulates the expression of NR4A1, CD14, and IL-1α within the MAPK signaling pathway, while increasing Arg-1 levels and promoting the secretion of IL-10. This collective shift in the inflammatory milieu favors an M2-like functional state in macrophages, an effect mediated through inhibition of the FOS/MAPK pathway, ultimately contributing to reduced plaque burden and ameliorated AS pathology. Furthermore, GP contributes to plaque stabilization by limiting lipid deposition and enhancing collagen fiber content (Chi et al., 2006; Ma et al., 2024). In ApoE^−/−^ mice, GP inhibits foam cell formation and accelerates AS regression, partly through effects on dendritic cell maturation (Liu L. et al., 2014; Liao et al., 2014). Overall, the collective preclinical evidence delineates a role for GP in curtailing atherosclerotic plaque development and reinforcing plaque stability through multi-target mechanisms. At present, the clinical evidence supporting the role of GP in AS is obviously insufficient. Although preclinical studies have elucidated its effects on signaling pathways and macrophage polarization, these mechanisms remain to be clinically validated. Future randomized controlled trials are needed to fill these key evidence gaps and to determine the efficacy and safety of GP in human subjects.

#### 5.1.9 Astragaloside IV

*Astragalus mongholicus* Bunge (Huangqi) has significant natural antioxidant activity and is effective in reducing the risk of AS (Wang T. et al., 2022). The main bioactive metabolites of Huangqi include polysaccharides, saponins, and flavonoids, among which AS-IV, a tetracyclic triterpenoid saponin (Zheng et al., 2020), is particularly important in cardiovascular protection due to its diverse pharmacological effects, including anti-inflammatory, antioxidant, anti-fibrotic, angiogenic, calcium-regulating, and lipid-lowering effects (Liang et al., 2023; Ou et al., 2023). Mitogen-activated protein kinase kinase 8 (MAP3K8), a key regulator of inflammation and immunity, plays a critical role in inflammation, immune regulation, endothelial function, and cell proliferation (Webb et al., 2019). These mechanistic insights indicate that AS-IV may act through multiple signaling pathways to regulate inflammation, oxidative stress, and endothelial function, thereby conferring protection against AS. Research by He et al. in ApoE^−/−^ mice elucidated that AS-IV reprograms macrophage polarization toward the M2 phenotype by targeting MAP3K8. This intervention resulted in suppressed MAP3K8 expression within the aortic tissue, concomitant inhibition of JNK and NF-κB p65 phosphorylation, and a marked upregulation of M2-associated markers (TGF-β, IL-4, IL-10, Arg-1). This phenotypic shift was further underpinned by the suppression of STAT1 signaling and potentiation of STAT6 activation (He et al., 2023). Beyond macrophage modulation, AS-IV activates PPAR-γ while concurrently suppressing the TLR4/NF-κB and PI3K/Akt pathways, actions that contribute to reduced lipid deposition and confer protection to endothelial cells (Zhang Y. et al., 2022). NRF2, a key antioxidant pathway, maintains redox balance by suppressing pro-inflammatory gene transcription (Lassègue et al., 2012; Kobayashi et al., 2016). In both *in vivo* AS rat models and *in vitro* ox-LDL-induced HUVEC models, AS-IV has been shown to activate NRF2—a central transcription factor in the antioxidant defense system—thereby alleviating oxidative stress. This activation contributes to the repair of oxidative stress-induced endothelial damage, suppresses the secretion of inflammatory factors, and ultimately confers therapeutic benefits against AS (Sun et al., 2018; Zhu et al., 2019). In summary, the collective preclinical evidence positions AS-IV as a multi-target agent capable of mitigating AS through coordinated immunomodulation, antioxidant effects, and endothelial protection. The generation of robust clinical evidence is therefore a pivotal next step for advancing AS-IV toward clinical application.

#### 5.1.10 Hesperidin

**FIGURE 3:**
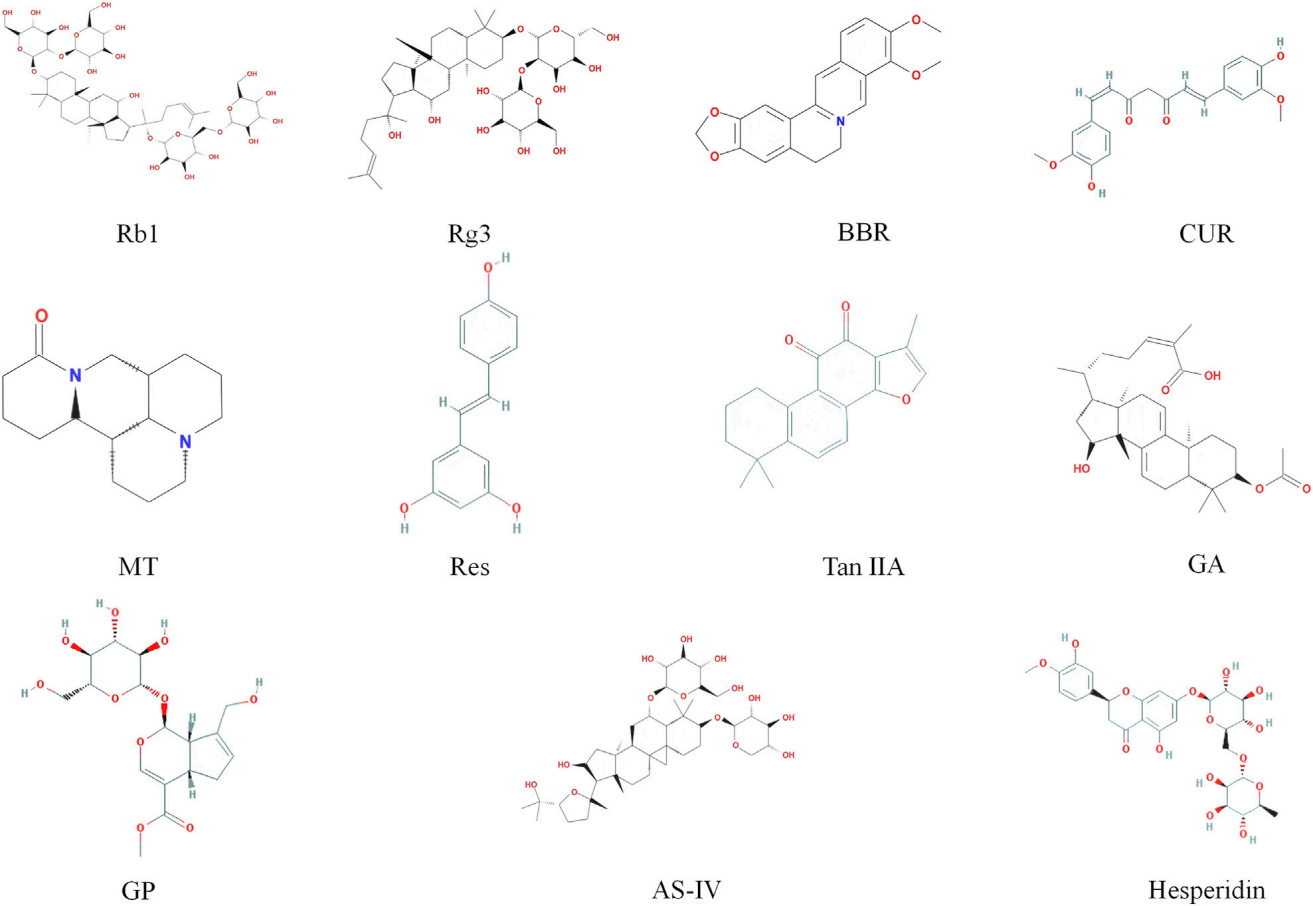
Chemical formula structure of the bioactive metabolites.

Hesperidin is a flavanone glycoside metabolite extracted from the mature fruit peel of citrus plants belonging to the Rutaceae family, such as orange (Citrus sinensis), grapefruit (Citrus paradise), and lemon (Citrus limon) (Pyrzynska, 2022). It is one of the most widely distributed plant phenolic metabolites in nature (Pyrzynska, 2022). Studies have shown that hesperidin exerts multifunctional pharmacological actions, including antioxidation, anti-inflammation, improvement of endothelial function, blood glucose regulation, and blood pressure reduction (Mas-Capdevila et al., 2020; Ortiz et al., 2022). These mechanisms indicate its potential role in cardiovascular protection and the prevention of AS. Investigations in ApoE^−/−^ mice and RAW264.7 macrophages have revealed that hesperidin activates the AMPK/PPAR-γ pathway and suppresses NF-κB (P65) expression, leading to a reprogramming of macrophage polarization towards an anti-inflammatory M2 phenotype (Fan et al., 2022). This shift in macrophage phenotype is associated with a favorable modulation of the inflammatory milieu, characterized by decreased secretion of TNF-α and IL-6 and increased expression of Arg-1 and IL-10. Consequently, hesperidin treatment reduces lipid deposition, inhibits high-fat diet-induced foam cell formation, and attenuates the development of atherosclerotic plaques (Mas-Capdevila et al., 2020; Fan et al., 2022). Cumulatively, these preclinical findings highlight hesperidin as a multi-target agent with significant potential to ameliorate cardiovascular risk factors and counteract atherosclerotic processes (Ebrahimi et al., 2023). Currently, clinical research on hesperidin primarily focuses on its impact on cardiovascular risk factors such as blood pressure, glycemic control, and systemic inflammation. Direct clinical evidence demonstrating its efficacy in intervening against atherosclerotic disease itself remains limited. While existing human data suggest potential cardiovascular benefits, large-scale, well-designed Randomized Controlled Trials are imperative to definitively establish its therapeutic value for AS. The chemical structure of the bioactive metabolite is shown in Figure 3. This review summarizes the signaling pathways related to the treatment of AS by bioactive metabolites by regulating macrophage polarization (Table 1).

**TABLE 1:** Signaling pathways related to the treatment of AS by bioactive metabolites by regulating macrophage polarization.

Signaling pathway	Mechanism of action	Bioactive metabolites	Types	Model	Dosage range	Macrophage polarization-related molecular targets		References
						Upregulation (M2)	Downregulation (M1)	
TLR4	TLR4/MyD88/NF-κB↓	BBR	Alkaloids	RAW264.7 cell	5, 10, 20 μmol/L	CD206	iNOS, IL-6, TNF-α	Li et al. (2020)
	TLR4/MAPK/NF-κB↓	CUR	Polyphenols	THP-1 cell	0, 7.5, 15, 30 μmol/L	-	TNF-α, IL-6, IL-12B (p40)	Zhou et al. (2015)
	TLR4/MyD88/NF-κB↓	GA	Tterpenoids	ApoE^−/−^ mice, BMDMs and RAW264.7 cell	*in vivo*: 1, 5, 25 mg/kg *in vitro*: 1, 5, 25 μg/mL	-	TNF-α, IL-6, IL-1β, MCP-1	Quan et al. (2024)
	TLR4/STAT1↓	MT	Alkaloids	M0-polarized primary mouse peritoneal macrophages	2.0 mmol/L	-	iNOS, TNF-α, IL-6, IL-1β	Cui et al. (2022)
NF-κB	TLR4/NF-κB/HIF-1α↓	Res	Polyphenols	RAW264.7 cell	1, 5, 10 μmol/L	-	IL-1β, IL-6, TNF-α	Guo et al. (2023)
	NF-κB↓	Tan IIA	Tterpenoids	ApoE^−/−^ mice	10 mg/kg/d	-	IL-6, TNF-α	Chen and Chen (2019)
	NF-κB (p65)↓	Hesperidin	Glycosides	ApoE^−/−^ mice, RAW264.7 cell	*in vivo*: 100, 200, 400 mg/kg *in vitro*: 5, 10, 20 μmol/L	-	TNF-α, IL-6	Fan et al. (2022)
MAPK	FOS/MAPK↓	GP	Glycosides	New Zealand white rabbits with HFD	1.5 mg/kg/d	IL-10, Arg-1	iNOS, IL-1β	Jin et al. (2020)
	MAP3K8↓	AS-IV	Glycosides	ApoE^−/−^ mice	20, 50 mg/kg	IL-10, IL-4, TGF-β, Arg-1	TNF-a	He et al. (2023)
PPAR-γ	PPAR-γ↑	20(S)-Rg3	Glycosides	Diabetic ApoE^−/−^ mice, BMDMs, RAW264.7 and THP-1 cell	*in vivo*: 10 mg/kg/2d *in vitro*: 25 μM	Arg-1, CD206	iNOS, CD86	Guo et al. (2018)
	AMPK/PPAR-γ↑	Hesperidin	Glycosides	ApoE^−/−^ mice, RAW264.7 cell	*in vivo*: 100, 200, 400 mg/kg *in vitro*: 5, 10, 20 μmol/L	IL-10, Arg-1	-	Fan et al. (2022)
JAK/STAT	p-STAT6↑	Rb1	Glycosides	ApoE^−/−^ mice, C57BL/6 mouse peritoneal macrophages	*in vivo*: 50 mg/kg/d *in vitro*: 10, 20, 40, 80 μM	IL-13, IL-4, IL-10, Arg-1, CD206	iNOS, MMP-9	Zhang X. et al. (2018)
	p-STAT6↑	BBR	Alkaloids	RAW264.7 cell	0, 5, 10, 25, 50, 100 μmol/L	-	iNOS, TNF-α, CXCL9	Chen YS. et al. (2024)
	p-STAT6↑	CUR	Polyphenols	RAW264.7 cell	0, 1, 10, 20, 40, 80 μmol/L	-	iNOS, TNF-α, CXCL9	Chen YS. et al. (2024)
	p-STAT6↑, p-STAT1↓	Tan IIA	Tterpenoids	ApoE^−/−^ mice	10 mg/kg/d	IL-10, TGF-β	IL-6, TNF-α	Chen and Chen (2019)

### 5.2 Herbal formulas

#### 5.2.1 Bu Yang Huan Wu decoction

Bu Yang Huan Wu Decoction (BYHW) was first recorded in 《Yi Lin Gai Cuo》 during the Qing Dynasty and was composed of seven botanical drug: Astragalus mongholicus Bunge. (Lamiaceae, Huangqin), Paeonia lactiflora Pall. (Paeoniaceae, Chishao), Carthamus tinctorius L. (Asteraceae, Honghua), Ligusticum chuanxiong S.H.Qiu, Y.Q.Zeng, K.Y.Pan, Y.C.Tang & J.M.Xu (Apiaceae, Chuanxiong), Juglans regia L. (Rosaceae, Taoren), Angelica sinensis (Oliv.) Diels (Apiaceae, Danggui), and Lumbricus (Dilong) (Mo et al., 2024). Modern research shows that BYHW provides vascular endothelial protection, regulates blood lipid levels, and exerts anti-AS effects through multi-metabolites and multi-target mechanisms, as well as widely used in the prevention and treatment of atherosclerotic disease, such as CVD (Zhang WW. et al., 2018). These pharmacological activities are largely attributed to its rich composition of bioactive metabolites, which act synergistically on inflammatory, lipid metabolic, and vascular pathways. It is important to note that while clinical application is widespread, the mechanistic understanding of BYHW’s anti-AS effects is predominantly derived from preclinical investigations. For example, in ApoE^−/−^ mice, BYHW inhibits the activation of the TLR4/MyD88/NF-κB signaling pathway, suppresses the expression of pro-inflammatory factors and adhesion molecules, inhibits foam cell formation, and promotes macrophage polarization toward the M2 phenotype (Li WY. et al., 2023). Bioactive metabolites in BYHW with anti-AS properties include AS-IV, paeoniflorin, ligustrazin, amygdalin, luteolin, ferulic acid, safflower yellow, and hydroxy-safflower yellow (Liu W. et al., 2022). Among these, glycosides reduce the release of pro-inflammatory factors and adhesion molecules and inhibit the phosphorylation of JAK2, STAT1, and STAT3, thereby alleviating AS inflammation (Fu et al., 2022). Further studies indicate that AS-IV, paeoniflorin, and amygdalin are among the principal bioactive metabolites in BYHW. These metabolites inhibit AS inflammation by suppressing the expression of STAT3, HIF-1, VEGF, and IκBα, as well as the nuclear translocation of NF-κB (Yan et al., 2023; Liu B. et al., 2020). Collectively, these findings suggest that the synergistic actions of multiple metabolites within BYHW converge on key inflammatory and lipid-regulating pathways, thereby attenuating atherosclerotic progression. BYHW is widely used in the clinical prevention and treatment of atherosclerotic diseases, such as cardiovascular disease. However, despite its long-standing application and anecdotal benefits, high-quality clinical trials specifically confirming its mechanisms, efficacy, and safety in AS remain limited.

#### 5.2.2 Zhuyu Pill

Zhuyu Pill (ZYP) was originally documented in 《Taiping Shenghuifang》 (Zhao Y et al., 2025), which was composed of Coptis deltoidea C.Y.Cheng & P.K.Hsiao (Ranunculaceae, Huanglian) and Tetradium ruticarpum (A.Juss.) T.G.Hartley (Rutaceae, Wuzhuyu) (Zhang X. et al., 2022). ZYP has effects on lowering blood lipids, reducing inflammation, and regulating glucose and lipid metabolism (Zhang X. et al., 2022). The main bioactive metabolites in ZYP are BBR, palmatine, evodiamine, and rutaecarpine (Pan et al., 2023). These metabolites exert synergistic actions on multiple signaling pathways, providing a mechanistic basis for ZYP’s anti-atherosclerotic activity. Mechanistic investigations indicate that ZYP improves AS by inhibiting the TLR4/MyD88/TRAF6 signaling pathway, activating STAT6 phosphorylation, and promoting M2-type macrophage polarization, thereby alleviating lipid metabolism disorders and inflammation (Zhao M. et al., 2025). Song et al. (2025) confirmed that ZYP regulates macrophage polarization, reduces foam cell and inflammatory cell aggregation, and lowers AS plaque deposition. Further studies found that in ApoE^−/−^ mice treated with ZYP, secretion of pro-inflammatory factors related to M1-type macrophages (TNF-α, IL-6) was significantly reduced, while the expression levels of NF-κB and iNOS were decreased. Conversely, anti-inflammatory factors associated with M2-type macrophages (IL-4, IL-13) were increased, along with the expression levels of PPAR-γ and Arg-1 (Song et al., 2024). By network pharmacology and *in vivo* experiments, ZYP was also found to exert anti-atherosclerotic effects through reduction of IL-6 and TNF-α levels and inhibition of the NF-κB pathway (Pan et al., 2023). Taken together, these findings suggest that ZYP attenuates AS progression via activation of the PPAR-γ/NF-κB signaling axis, suppressing M1 macrophage polarization while promoting M2 differentiation. This results in reduced inflammatory infiltration within arterial tissues and inhibition of plaque formation. Recent clinical trials have begun to evaluate ZYP’s efficacy in patients with hyperlipidemia complicated by carotid AS (Song et al., 2025). While preliminary data are encouraging, mechanistic insights remain predominantly derived from preclinical models, and high-quality randomized controlled trials directly confirming its anti-atherosclerotic effects are still lacking.

#### 5.2.3 ZeXieYin formula

ZeXieYin formula (ZXYF), which can be traced back to 《Huangdi Neijing》, is formulated with three specified botanical drugs: Alisma plantago-aquatica L. (Alismataceae, Zeie), Atractylodes macrocephala Koidz. (Asteraceae, Baizhu), and Pyrola calliantha Andres. (Ericaceae, Luxiancao) (Sun et al., 2024). It is known for its ability to regulate lipid metabolism (Zhu et al., 2020). The traditional use of ZXYF in lipid regulation provides a theoretical foundation for its potential application in the prevention and treatment of AS. Huang Y et al. (2024) demonstrated in ApoE^−/−^ mice that ZXYF significantly reduces inflammation by inhibiting the activation of ERK and p38 in the MAPK family and the expression of p-NF-κB. It also reduces the area of the necrotic lipid core and promotes macrophage polarization toward the M2 phenotype. Additionally, ZXYF inhibits the secretion of MMPs, increases plaque stability, and prevents plaque rupture (Huang R. et al., 2024). ZXYF also modulates the PI3K/Akt/SREBP-1 signaling pathway, reducing the levels of inflammatory cytokines (e.g., IL-1β, TNF-α, IL-10, IL-17, and IL-6) in serum, thereby slowing the progression of AS (Liu R. et al., 2023). Together, these animal studies indicate that ZXYF exerts anti-atherosclerotic effects through multi-target modulation of inflammatory signaling, macrophage polarization, and plaque stabilization. The evidence supporting the use of ZXYF in AS is currently limited to animal studies. Although ZXYF has a long history of clinical use for metabolic regulation in traditional medicine, there is a lack of direct clinical trials confirming its efficacy, mechanisms, and safety in patients with AS.

#### 5.2.4 Qingre Huoxue Decoction

Qingre Huoxue Decoction (QRHX) has been clinically applied for more than two decades, is composed of seven botanical drugs: Astragalus mongholicus Bunge. (Lamiaceae, Huangqin), Paeonia lactiflora Pall. (Paeoniaceae, Chishao), Ilex pubescens Hook. & Arn. (Aquifoliaceae, Maodongqing), Carthamus tinctorius L. (Asteraceae, Honghua), Ligusticum chuanxiong S.H.Qiu, Y.Q.Zeng, K.Y.Pan, Y.C.Tang & J.M.Xu (Apiaceae, Chuanxiong), Dalbergia odorifera T.C.Chen (Fabaceae, Jiangxiang), and Salvia miltiorrhiza Bunge. (Lamiaceae, Danshen) (He W. et al., 2024). QRHX contains high levels of baicalin and salvianolic acid B, both of which exert anti-AS effects by regulating the NF-κB signaling pathway and macrophage polarization (He W. et al., 2024). The multi-metabolite and multi-target nature of QRHX provides a mechanistic basis for its anti-inflammatory, lipid-regulating, and plaque-stabilizing activities. In an *in vivo* study on ApoE^−/−^ mice, QRHX induces macrophage polarization toward the M2 phenotype by inhibiting the NF-κB signaling pathway, thereby alleviating plaque inflammation. Additionally, QRHX targets and inhibits key proteins in the PI3K/Akt signaling pathway, promotes M2 polarization, and reduces the expression of M1 phenotype-related inflammatory factors (TNF-α, MCP-1, and NLRP3), exerting anti-inflammatory, lipid-regulating, and plaque-stabilizing effects (Jin, 2021). Furthermore, QRHX increases miR-26a-5p expression, decreases PTGS2 expression, and promotes the remodeling of M1-type macrophages to the M2 phenotype, thereby improving AS pathology (He W. et al., 2024). These preclinical findings provide biological plausibility for the clinical effects observed in patients and illustrate a translational pathway from mechanistic studies to therapeutic application. QRHX has demonstrated efficacy in the treatment of AS, showing potential to stabilize plaques and reduce the incidence of adverse cardiovascular events, based on evidence from clinical studies (Zuo et al., 2021). Importantly, QRHX is one of the few traditional formulas for which both clinical and preclinical evidence converge, with clinical observations guiding mechanistic investigations in animal models. This review summarizes the signaling pathways related to the treatment of AS by herbal formulas by regulating macrophage polarization (Table 2).

**TABLE 2:** Signaling pathways related to the treatment of AS by herbal formulas by regulating macrophage polarization.

Signaling pathway	Mechanism of action	Herbal formulas name	Botanical drugs	Model	Dosage range	Macrophage polarization-related molecular targets		References
						Upregulation (M2)	Downregulation (M1)	
TLR4	TLR4/MyD88/NF-κB↓	BYHW	Astragalus mongholicus, Paeonia lactiflora, Carthamus tinctorius, Ligusticum chuanxiong, Juglans regia, Angelica sinensis, and Lumbricus	ApoE^−/−^ mice	2.772g/kg/d	-	TNF-α, IL-6, VCAM-1	Li WY. et al. (2023)
NF-κB	NF-κB↓	QRHX	Astragalus mongholicus, Paeonia lactiflora, Ilex pubescens, Carthamus tinctorius, Ligusticum chuanxiong, Dalbergia odorifera, and Salvia miltiorrhiza	ApoE^−/−^ mice, RAW264.7 cell	*in vivo*: 7.5, 15, 30 g/kg/d *in vitro*: 0, 1, 2.5, 5, 10, 20, 50, 100 μg/mL	Arg-1, CD163, IL-4, IL-10	iNOS, TNF-α, MCP-1	Jin Z. et al. (2023)
MAPK	MAPK/NF-κB↓	ZXYF	Alisma plantago-aquatica, Atractylodes macrocephala, and Pyrola calliantha	ApoE^−/−^ mice	3.8 g/kg/d	IL-10	IL-1α, IL-1β, IL-6, TNF-α	Huang R. et al. (2024)
PPAR-γ	PPAR-γ/NF-κB↑	ZYP	Coptis deltoidea and Tetradium ruticarpum	ApoE^−/−^ mice	130.54, 261.08, 522.16 mg/kg/d	Arg-1, IL-13, IL-4	iNOS, TNF-α, IL-6	Song et al. (2024)
PI3K/Akt	PI3K/Akt↓	QRHX	Astragalus mongholicus, Paeonia lactiflora, Ilex pubescens, Carthamus tinctorius, Ligusticum chuanxiong, Dalbergia odorifera, and Salvia miltiorrhiza	ApoE^−/−^ mice	7.5, 15, 30 g/kg/d	Arg-1	iNOS, TNF-α, MCP-1, NLRP3	Jin. (2021)

## 6 Discussion

AS is a serious threat to the health of the human cardiovascular system and is closely associated with a variety of acute cardiovascular events. The pathogenesis of AS is complex, involving the interaction of endothelial dysfunction, imbalance of lipid metabolism, and blood flow abnormalities, among which the inflammatory response is the core mechanism driving plaque formation and development. Macrophages are an important part of the human immune function and are involved in the inflammatory response *in vivo*, and they can be transformed into different cell subtypes according to different environments, which is an important manifestation of macrophage plasticity. Macrophages can be categorized into pro-inflammatory M1-type macrophages and anti-inflammatory M2-type macrophages based on differences in macrophage activation pathways and functions. Macrophage phenotypic differentiation has an important impact on AS regression. Early AS plaques are predominantly M2-type macrophages, but with the progression of the disease, the proportion of M1-type macrophages increases significantly and triggers vascular events, indicating that the regulation of the dynamic balance of M1/M2 polarization is a key link in inhibiting AS plaque formation and stabilizing plaques. Therefore, directing M1-type macrophages to M2-type macrophages has become a novel therapeutic strategy to alleviate the pathological process of AS.

Medical treatment has now entered the era of precision medicine. With the development of systems biology and network pharmacology, the molecular mechanisms of Chinese medicine in regulating macrophage polarization-related signaling pathways have gradually been clarified, providing a scientific basis for constructing a precision treatment system that combines Chinese and Western medicine. Exploring the role of Chinese medicine in precisely targeting macrophage polarization via signaling pathways and identifying new therapeutic targets for the prevention and treatment of AS are of great clinical significance. This study systematically reviews the mechanisms by which bioactive metabolites and herbal formulas ameliorate AS through the regulation of macrophage polarization multiple signaling pathways. Several metabolites, including BBR, CUR, Tan IIA, Rb1/Rg3, and GA, as well as the herbal formulas BYHW and ZYP, promote a shift toward the M2 macrophage phenotype, thereby enhancing plaque stability. Accumulated evidence indicates that suppression of the TLR4/MyD88/NF-κB and MAPK pathways, coupled with activation of the PPAR-γ/STAT6 axis, serves as a central mechanism underlying macrophage modulation by these interventions. These findings have been consistently validated in ApoE^−/−^ mouse models as well as in RAW264.7 and THP-1 cellular assays. In contrast, the roles of the PI3K/Akt and certain branches of the MAPK pathway in regulating macrophage polarization within the AS microenvironment remain inconclusive and merit further investigation to elucidate their context-dependent functions (Zhao Y. et al., 2021).

AS is a major pathological basis of CVD, and the preventive and therapeutic effects of TCM on AS contribute to slowing CVD progression. Clinical trials have demonstrated that TCM exerts broad pharmacological actions in alleviating CVD symptoms with a favorable safety profile. Evidence indicates that, compared with conventional Western medicine alone, adjunctive TCM interventions significantly improve treatment outcomes for metabolic syndrome metabolites such as hypertension. High-quality, multicenter randomized controlled trials have further shown that Tongxinluo reduces carotid plaque progression and lowers the risk of major adverse cardiovascular and cerebrovascular events (MACE). Shexiang Baoxin Pill alleviates angina symptoms and reduces the incidence of MACE (Ge et al., 2021). In addition, bioactive metabolites—such as BBR, CUR, and Rb1—not only demonstrate clear efficacy in treating CVD but also are associated with a low incidence of adverse reactions. Overall, from bioactive metabolites to herbal formulas, the growing body of evidence supports the multitarget potential of TCM in the prevention and treatment of CVD.

Rooted in classical TCM theory, the concept of “medicine–food homology” (Yao Shi Tong Yuan) posits that it refers to substances with both nutritional and medicinal value under TCM theory, botanical drugs like Hawthorn are commonly used in both contexts. Consequently, numerous bioactive metabolites derived from these sources hold promise as both therapeutic candidates for CVD and dietary supplements. Preclinical and clinical studies have demonstrated that BBR exerts potent anti-AS effects and significantly improves parameters related to metabolic syndrome (McCubrey et al., 2017). Similarly, CUR has been shown to reduce multiple AS risk factors in large-scale randomized controlled trials, establishing it as an ideal candidate for functional food development (Yaikwawong et al., 2024; Xu et al., 2018). Beyond its nutraceutical benefits, CUR also serves as a natural edible pigment, attracting considerable interest from both the scientific community and the public (Sharifi-Rad et al., 2020). Res has garnered clinical support for its advantages in cardiometabolic protection and lipid regulation (Parsamanesh et al., 2021), with additional promising applications in nutraceuticals, including anti-osteoporosis, anti-vascular aging, and anti-obesity interventions (Cao et al., 2022). Hesperidin mitigates AS by improving endothelial function and modulating lipid profiles; however, its oral bioavailability remains limited. Consumption in the form of citrus juices or specialized supplements may enhance its absorption (Testai and Calderone, 2017; Shylaja et al., 2024; Salden et al., 2016). Hesperidin is also considered a promising dietary supplement. It has been shown to improve muscle metabolic status, thereby facilitating functional recovery and enhancing exercise performance (Martínez-Noguera et al., 2019). Collectively, these bioactive metabolites are increasingly transcending cultural and regional boundaries, gaining recognition within Western medical and nutritional sciences. This trend not only amplifies the global relevance of TCM but also reflects sustained international research interest in the multifunctional potential of natural bioactive metabolites.

As of the prespecified search date, variability in randomization, blinding, sample-size justification, endpoint selection, and reporting transparency persists across studies. Clinical trials are often underpowered with limited follow-up, and many *in vitro* or single-animal studies lack external calibration against human data. These issues impede comparability and raise risks of bias. These issues impede comparability and raise risks of bias. While *in vitro* systems are valuable for hypothesis generation and mechanistic testing, their lack of integrated metabolic–immune–multi-organ context warrants cautious interpretation and cross-validation in animal and clinical settings. Substantial variability in extraction procedures, experimental designs, and outcome assessment criteria contributes to pronounced heterogeneity, thereby limiting cross-study comparability and the reliability of integrative analyses. Heterogeneity arises from four domains: 1) models (species, sex, age, comorbidities, diet/induction); 2) interventions/formulations (composition, extraction, chemical fingerprints, active-content consistency); 3) endpoints/assays (definitions and measurements for inflammation, lipid handling, plaque stability); and 4) analytical frameworks (pathway selection and depth of causal validation). Limited incorporation of TCM-specific constructs (e.g., pattern differentiation) into animal models further constrains generalizability.

Although TCM shows considerable potential for the treatment of AS-related diseases, its translation from basic research to clinical application remains fraught with challenges. Bench-to-bedside translation is limited by insufficient pharmacokinetics/toxicology and organ-level safety data, potential synergy/antagonism within multi-herb formulations and batch-to-batch variability. Many of the bioactive metabolites discussed herein, such as CUR and Res, have been identified as ‘pan-assay interfering compounds’ (PAINS) (Magalhães, et al., 2021; Bolz, et al., 2021). This class of compounds may generate false-positive results in in vitro biochemical or cell-based high-throughput screens through non-specific mechanisms, such as redox activity, protein aggregation, fluorescence interference, or membrane disruption (Magalhães, et al., 2021). Consequently, conclusions drawn from *in vitro* assays, particularly those involving multi-target effects, must be interpreted with extreme caution. Although this review aims to map the network of their potential mechanisms of action rather than directly advocate for clinical translation, we must emphasize that these *in vitro* findings at best constitute a hypothetical foundation for subsequent research. A significant gap remains between these findings and genuine pharmacological activity or ultimate clinical relevance.

Future studies should integrate *in vitro* systems, animal models, and clinical data to minimize species-dependent limitations, exclude potential confounding effects, and verify the true biological activity of candidate metabolites, thereby enhancing the clinical relevance and translational value of the findings. To overcome current limitations, future efforts should adopt multidisciplinary approaches and establish standardized frameworks to promote the precision and internationalization of TCM-based strategies for AS prevention and therapy. Key directions include:1) Improving Study Quality and Methodological Standardization: Implement rigorous randomization, blinding, allocation concealment, and transparent reporting; develop internationally recognized quality-control standards, experimental design guidelines, and outcome evaluation frameworks to strengthen study rigor and reproducibility. 2) Reducing Heterogeneity and Enhancing Quality Control: Standardize herbal formulation quality, extraction procedures, and dosing regimens, and define consistent outcome measures to minimize inter-study variability and facilitate multicenter data integration and meta-analyses. 3) Optimizing Clinical Translation and Personalized Therapy: Increase sample sizes, extend follow-up durations, incorporate real-world evidence, and include participants across different ages, sexes, and comorbidity profiles. Integrate patient stratification, genetic background, and metabolic characteristics to explore individualized treatment strategies and enhance generalizability and clinical utility. 4) Multidimensional Evidence Integration and Technological Innovation: Combine *in vitro*, animal, and clinical investigations, and leverage multi-omics, network pharmacology, and artificial intelligence–based approaches for mechanistic validation and efficacy prediction, thereby improving drug discovery efficiency. Collectively, these strategies are expected to standardize methodologies, improve research quality and reproducibility, reduce heterogeneity, and—together with cutting-edge technologies—drive the development of safer, more effective, and evidence-based TCM interventions, providing a robust scientific foundation for precision prevention and treatment of AS.

## Glossary

AGEs: advanced glycation end-products
Akt: protein kinase B
AMPK: AMP-activated protein kinase
**ApoE** ^**−/−**^: apolipoprotein E knockout
Arg-1: arginase-1
AS: atherosclerosis
AS-IV: Astragaloside IV
BBR: Berberine
BYHW: Bu Yang Huan Wu Decoction
CUR: Curcumin
CVD: cardiovascular diseases
ECM: extracellular matrix
ECs: endothelial cells
ERK: extracellular signal-regulated kinase
GA: Ganoderic acid
G. lucidum: Ganoderma lucidum
GP: Gardenia glycoside
HFD: high-fat diet
HIF-1α: hypoxia-inducible factor
IFN-γ: interferon-gamma
IκB: inhibitory κB
IKK: IκB kinase
IL: interleukin
iNOS: inducible nitric oxide synthase
JAK: Janus kinase
JNK: c-Jun N-terminal kinase
LPS: lipopolysaccharide
MAPK: mitogen-activated protein kinase
MAP3K8: mitogen-activated protein kinase kinase kinase 8
MMPs: matrix metalloproteinases
MT: Matrine
NF-κB: nuclear factor-kappa B
NLRP3: NOD-like receptor protein 3
Nrf2: Nuclear factor E2-related factor 2
ox-LDL: oxidized low-density lipoprotein
PAMPs: pathogen-associated molecular patterns
PI3K: phosphoinositide 3-kinase
PPAR-γ: peroxisome proliferator-activated receptor γ
QRHX: Qingre Huoxue Decoction
Rb1: Ginsenoside Rb1
Res: Resveratrol
Rg3: Ginsenoside Rg3
ROS: reactive oxygen species
SMCs: smooth muscle cells
STAT: signal transducer and activator of transcription
Tan IIA: Tanshinone IIA
TCM: traditional Chinese medicine
TGF-β: transforming growth factor-β
Th1: T Helper 1
Th2: T Helper 2
TLR4: Toll-like receptor 4
TLRs: Toll-like receptors
TNF-α: tumor necrosis factor-alpha
TRAF6: tumor necrosis factor receptor-associated factor 6
VEGF: vascular endothelial growth factor
ZXYF: ZeXieYin formula
ZYP: Zhuyu Pill
